# Cytotoxic Activity of Curcumin- and Resveratrol-Loaded Core–Shell Systems in Resistant and Sensitive Human Ovarian Cancer Cells

**DOI:** 10.3390/ijms26010041

**Published:** 2024-12-24

**Authors:** Joanna Weżgowiec, Zofia Łapińska, Łukasz Lamch, Anna Szewczyk, Jolanta Saczko, Julita Kulbacka, Mieszko Więckiewicz, Kazimiera A. Wilk

**Affiliations:** 1 Department of Experimental Dentistry, Wroclaw Medical University, 50-425 Wroclaw, Poland; joanna.wezgowiec@umw.edu.pl; 2 Department of Molecular and Cellular Biology, Wroclaw Medical University, 50-556 Wroclaw, Poland; zofia.lapinska@student.umw.edu.pl (Z.Ł.); a.szewczyk@umw.edu.pl (A.S.); jolanta.saczko@umw.edu.pl (J.S.); julita.kulbacka@umw.edu.pl (J.K.); 3 Department of Engineering and Technology of Chemical Processes, Wrocław University of Science and Technology, Wybrzeże Wyspiańskiego 27, 50-370 Wrocław, Poland; lukasz.lamch@pwr.edu.pl (Ł.L.); kazimiera.wilk@pwr.edu.pl (K.A.W.); 4 Department of Immunology and Bioelectrochemistry, State Research Institute Centre for Innovative Medicine, Santariškių g. 5, LT-08406 Vilnius, Lithuania

**Keywords:** phytopharmaceuticals, curcumin, resveratrol, anticancer agents, drug carriers, nanocarriers, polymeric nanoparticles, drug delivery systems, encapsulation, ovarian adenocarcinoma

## Abstract

Due to the high mortality rate of ovarian cancer, there is a need to find novel strategies to improve current treatment modalities. Natural compounds offer great potential in this field but also require the careful design of systems for their delivery to cancer cells. Our study explored the anticancer effects of novel resveratrol (RSV)- and curcumin (CUR)-loaded core–shell nanoparticles in human ovarian cancer cells. We evaluated the in vitro cytotoxicity of various nanocarriers (CUR 1-3, RSV I-III) delivered to MDAH-2774 and SKOV-3 cells in comparison to free RVS and CUR after 24 h and 72 h treatment. A two-way ANOVA was applied to compare the results of the MTT assay. Confocal laser scanning microscopy was employed to visualize cellular uptake and mitochondrial localization. Our findings revealed that the cytotoxicity of the core–shell nanoparticles with RSV was not significant, but the systems loaded with CUR effectively decreased the viability of cells. The MDAH-2774 cell line was more sensitive to the treatment than SKOV-3. The enhanced cellular uptake of CUR delivered by core–shell systems and its colocalization with mitochondria were demonstrated. Further research focused on the detailed biological effects of the most effective systems (CUR 2 and CUR 3) should be conducted to provide detailed insights. These findings highlight the promising role of CUR-loaded nanoparticles in ovarian cancer treatment.

## 1. Introduction

Ovarian cancer, being the eighth most common cancer in women, accounts for an estimated 4.7% of cancer deaths in 2020 [1]. Currently, surgery followed by cisplatin- and paclitaxel-based chemotherapy are the recommended treatments for this gynecological malignancy [2]. Unfortunately, it is still the leading cause of cancer-related mortality among women due to a late diagnosis due to only subtle symptoms occurring before the advanced stage of the tumor and a high recurrence rate (80%) followed by drug resistance [3]. Thus, the high mortality rate of ovarian cancer remains a huge clinical challenge, and, therefore, it is necessary to find a strategy for the improved treatment of this disease [4,5].

One of the approaches used to fight against ovarian cancer is based on natural bioactive compounds. Such substances attract the attention of many research groups due to the broad spectrum of their biological activity, lack of or limited side effects, and ease of accessibility [6,7,8,9]. In particular, resveratrol (RSV) and curcumin (CUR) are some of the most intensively investigated compounds among numerous phytopharmaceuticals. Resveratrol is a polyphenol produced by many different plants, including grapes, blueberries, cranberries, and peanuts. It has antioxidative, anti-inflammatory, cardioprotective, neuroprotective, and anticancer properties [10,11,12]. The mechanisms of the anticancer effect of resveratrol are related to damage to mitochondrial function, increased production of reactive oxygen species (ROS), and apoptosis. Curcumin is a polyphenol that originates from the turmeric plant (*Curcuma longa*), possessing anti-inflammatory, antioxidative, immunoregulatory, antifungus, antibacterial, and antitumor properties. Mechanisms of its anticancer activity involve affecting several signaling pathways related to apoptosis, autophagy, epithelial–mesenchymal transition (EMT), angiogenesis, proliferation, and metastasis [13,14,15,16,17].

Despite the numerous beneficial effects of both resveratrol and curcumin, the clinical applicability of these polyphenols is still limited due to their poor water solubility, chemical instability, rapid metabolism, and low bioavailability [18]. Various approaches have been proposed to overcome these problems, including the development of derivatives or analogs with improved therapeutic efficacy [19,20,21,22,23,24,25] and the design of novel drug delivery systems. Huge attention is paid to nanotechnology for enhancing the delivery and stability of natural compounds used for the treatment of ovarian cancer [14,26,27,28,29]. Various kinds of nano-carriers could be used for drug deliveryf to ensure a controlled drug release rate. Among multiple classes of nanoparticles, core–shell is a particularly promising structure with numerous biomedical applications. Such nanoparticles combine core structures, which can be used for the encapsulation of poorly water-soluble materials with shells, which may serve to anchor bioactive agents and contain hydrophilic compounds, stabilizing the whole platform and preventing nanoparticle aggregation [30,31,32]. Due to tunable properties, core–shell nanosystems are a promising platform for the delivery of natural compounds to ovarian cancer cells. However, the fabrication of such nanoparticles and their properties still require investigation.

The present research aimed to explore the anticancer effect of novel resveratrol-loaded and curcumin-loaded core–shell nanoparticles delivered to human ovarian cancer cells. The systems were designed and characterized in a previous study by Lamch using a membrane-assisted approach to provide an efficient technique for the encapsulation of curcumin [32] and resveratrol [31]. In the current study, we evaluated the in vitro cytotoxicity of such nanocarriers in human ovarian cancer cells. Finally, the identification of the platforms with the highest potential paved the way for further investigation aimed at improving ovarian cancer treatment outcomes.

## 2. Results

### 2.1. Physicochemical Characterization

Our systems (see Table 1 and Figures S1 and S2 in ESI) were characterized by hydrodynamic diameters from around 120-150 nm (systems stabilized by PSS-MA derivatives with ester linkages between the backbone and the hydrophobic chains) to ca 250 nm (system stabilized by PSS-MA derivatives with ester linkages between the backbone and the hydrophobic chains). Such features were in good agreement with the designed and optimized systems [31,32], showing the high potential and excellent reproducibility of the membrane-assisted properties. Moreover, all samples were characterized by PdIs between ca 0.07 and 0.16, corresponding with highly uniform size distribution.

The concentrations of active payloads, i.e., curcumin and resveratrol, were tuned to determine the influence of core-forming and shell-forming materials. In general, the concentration of RSV in the core–shell nanoparticles’ dispersions (ca 325–400 μM) was around one order of magnitude higher than CUR (ca 25–55 μM). The detailed methodology for determining RSV and CUR concentration is described in Section 4.3 in the Materials and Methods section, while the calibration curves for the acetone/water (5:1, *v*:*v*) mixtures are shown in Figures S3 and S4 in ESI. Further information is also available in publications [31,32], where optimization studies for our systems are presented. Taking into account the optimized preparation methodologies, it was possible to introduce any amount of the active payload lower or equal to the optimal/maximal one since the influence of core-forming and shell-forming polymers on samples’ dimensions and stability was much more significant when compared with the active payloads. Our systems were characterized by high encapsulation efficiency (*EE%*) values, exceeding 65%, corresponding with the intended drug loading (*DL%*) contents.

Samples’ visualization by AFM microscopy confirmed DLS data: low polydispersity and mean diameters not exceeding values by size distribution (maximal height at Z scales around 190–210 nm) and smaller nanoparticles for the CUR loaded system. Moreover, it clearly showed the nearly spherical shape of the core–shell nanoparticles, corresponding to high values of zeta potential (<−70 mV, negative charge due to the utilized polyelectrolytes), assuming excellent stability (Figures S1 and S2) (see [31,32]).

Our considerations were supported by calculations of solubility parameter differences for CUR-PLLA and RSV-PLGA pairs—see Table S1 in ESI. Since we needed to compare systems of relatively high molecular weights (>200 Da), our calculations possessed only relative meaning, i.e., they could distinguish which system was more thermodynamically compatible. The calculated values for CUR-PLLA and RSV-PLGA pairs were given by numbers 15.0 MPa^0.5^ and 30.1 MPa^0.5^, respectively.

### 2.2. Cytotoxicity

**Figure 1:**
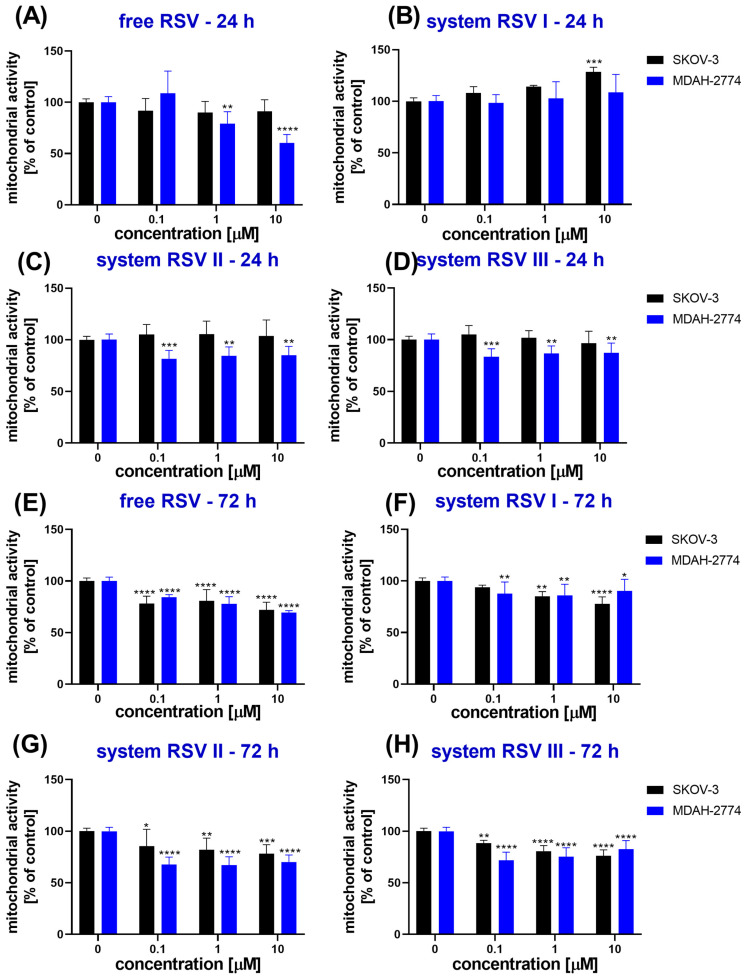
The cytotoxicity of free resveratrol (**A**,**E**) and resveratrol loaded in the core–shell systems (**B**–**D**,**F**–**H**), evaluated using an MTT assay based on the mitochondrial activity after 24 h (**A**–**D**) and 72 h (**E**–**H**) in SKOV-3 and MDAH-2774 ovarian cancer cell lines; * *p* < 0.05, ** *p* < 0.01, *** *p* < 0.001, **** *p* < 0.0001.

In the studied range of concentrations, both free RSV and the core–shell systems designed (RSV I, II, III) induced no cytotoxic effect in the SKOV-3 cell line after 24 h of incubation (Figure 1A–D). The MDAH-2774 ovarian cancer cell line was more sensitive to the treatment, revealing a decrease in cellular viability, particularly after incubation with the RSV II and RSV III systems (Figure 1C,D). Moreover, free RSV also significantly impaired the mitochondrial function of cells, reducing it to 60% when compared with the untreated control cells (Figure 1A). In contrast, the RSV I system was not cytotoxic to both cell lines; for SKOV-3 cells, the metabolic activity increased slightly upon 24 h of contact with these nanoparticles (Figure 1B).

Evaluation of the cellular viability changes due to prolonged contact with the studied systems showed that the effects observed were not temporary (Figure 1E–H). After 72 h of incubation, the mitochondrial activity was lowered, and the strongest reduction was observed for RSV II (ca. 67% of the untreated control cells) (Figure 1G). Similar to the findings revealed after 24 h, the weakest cytotoxic effect was induced by the RSV I system (Figure 1F), and the SKOV-3 cell line was more resistant to RSV than the MDAH-2774 cell line.

The calculation of IC_50_ confirmed the more pronounced cytotoxicity of RSV to the MDAH-2774 than the SKOV-3 cells (Table 2). It was also revealed that the longer time of exposure of cells to free RSV or the studied systems resulted in a stronger inhibition of cellular viability as the IC_50_ values were decreased. For the SKOV-3 cell line, the lowest IC_50_ was obtained for RSV III, while, for the MDAH-2774 cell line, the RSV II system induced the strongest cytotoxicity. However, the decrease in viability achieved was still similar to or even milder than that with free RSV.

**Figure 2:**
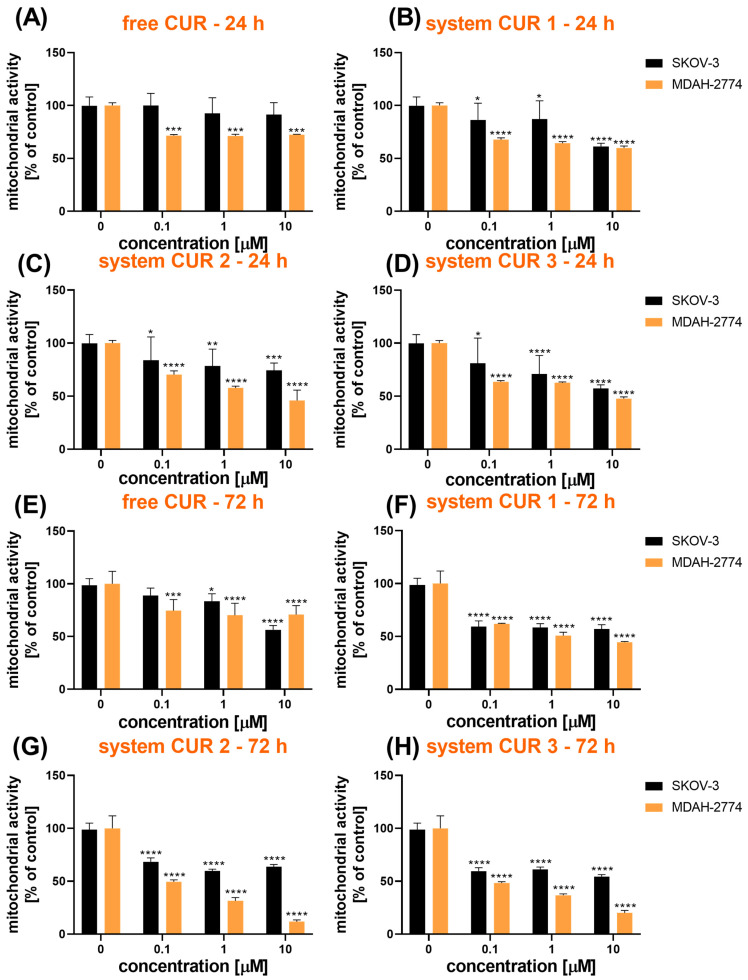
The cytotoxicity of free curcumin (**A**,**E**) and curcumin loaded in the core–shell systems (**B**–**D**,**F**–**H**), evaluated using MTT assay based on the mitochondrial activity after 24 h (**A**–**D**) and 72 h (**E**–**H**) in SKOV-3 and MDAH-2774 ovarian cancer cell lines; * *p* < 0.05, ** *p* < 0.01, *** *p* < 0.001, **** *p* < 0.0001.

The second phytochemical—curcumin—induced a stronger cytotoxic effect in ovarian cancer cells (Figure 2). For MDAH-2774 cells, free CUR reduced the metabolic activity to ca. 71% of the untreated control (Figure 2A). Moreover, the entrapment of CUR in the core–shell systems resulted in an additional decrease in cellular viability. The strongest cytotoxicity was induced upon treatment with CUR 2 (46% of the untreated control) (Figure 2C) and CUR 3 (48% of the untreated control) (Figure 2D). Interestingly, the effects observed in SKOV-3 were slighter than in MDAH-2774, but, due to the loading of CUR in the core–shell particles, a significant reduction in viability was achieved in this cell line.

The effects observed after 72 h confirmed the strong cytotoxicity of the core–shell systems loaded with CUR, particularly CUR 2 and CUR 3 (Figure 2E–H). For MDAH-2774 cells, the lowest level of mitochondrial activity was revealed for CUR 2 (12% of untreated control) and CUR 3 (20% of untreated control) at 10 µM (Figure 2G,H). The SKOV-3 cell line was more resistant to the treatment, but a significant reduction in viability was also achieved. The strongest effect occurred for CUR 3 (54% of the untreated control) and CUR 1 (57% of the untreated control) (Figure 2H,F).

The calculation of IC_50_ confirmed that longer exposure of cells to free CUR or the studied core–shell systems resulted in a stronger inhibition of cellular viability as the IC_50_ values were decreased (Table 3). More pronounced cytotoxicity of CUR to the MDAH-2774 than the SKOV-3 cells was also revealed. For the SKOV-3 cell line, the lowest IC_50_ was obtained for CUR 3, while, for the MDAH-2774 cell line, the CUR 2 system induced the strongest cytotoxicity. Similar conclusions were obtained for the systems loaded with RSV (Table 2). Moreover, the study of CUR revealed that due to the entrapment of this phytopharmaceutical in the core–shell particles, a significant decrease in the viability of ovarian cancer cells could be achieved.

### 2.3. Confocal Laser Scanning Microscopy (CLSM) Visualization of Cells and Intracellular Localization of CUR

**Figure 3:**
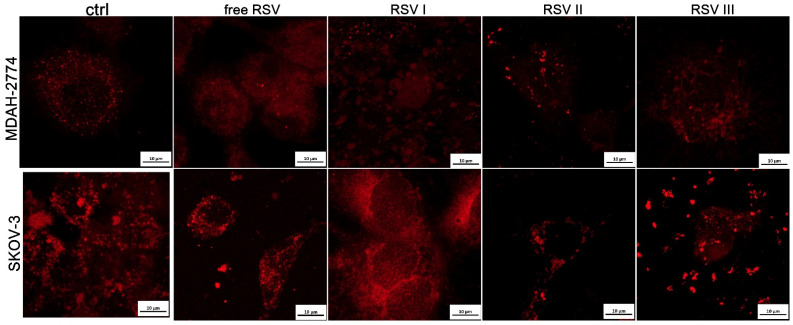
Confocal laser scanning microscopy (CLSM) visualization of mitochondria (in red) after 24 h of incubation of SKOV-3 and MDAH-2774 ovarian cancer cells with free resveratrol and resveratrol loaded in the core–shell systems.

Alternations in mitochondrial distribution were observed in ovarian cancer cells after incubation with RSV (Figure 3). In particular, MDAH-2774 cells were more susceptible to the cytotoxic influence of RSV. In untreated control cells, mitochondria formed dense networks around the nuclei, while, in the cells treated with RSV, they were distributed in more unstructured, scattered clusters. For SKOV-3 cells, the alterations induced in mitochondria were less visible. However, the core–shell system RSV III induced the most significant changes in mitochondria distribution in this cell line (Figure 3).

**Figure 4:**
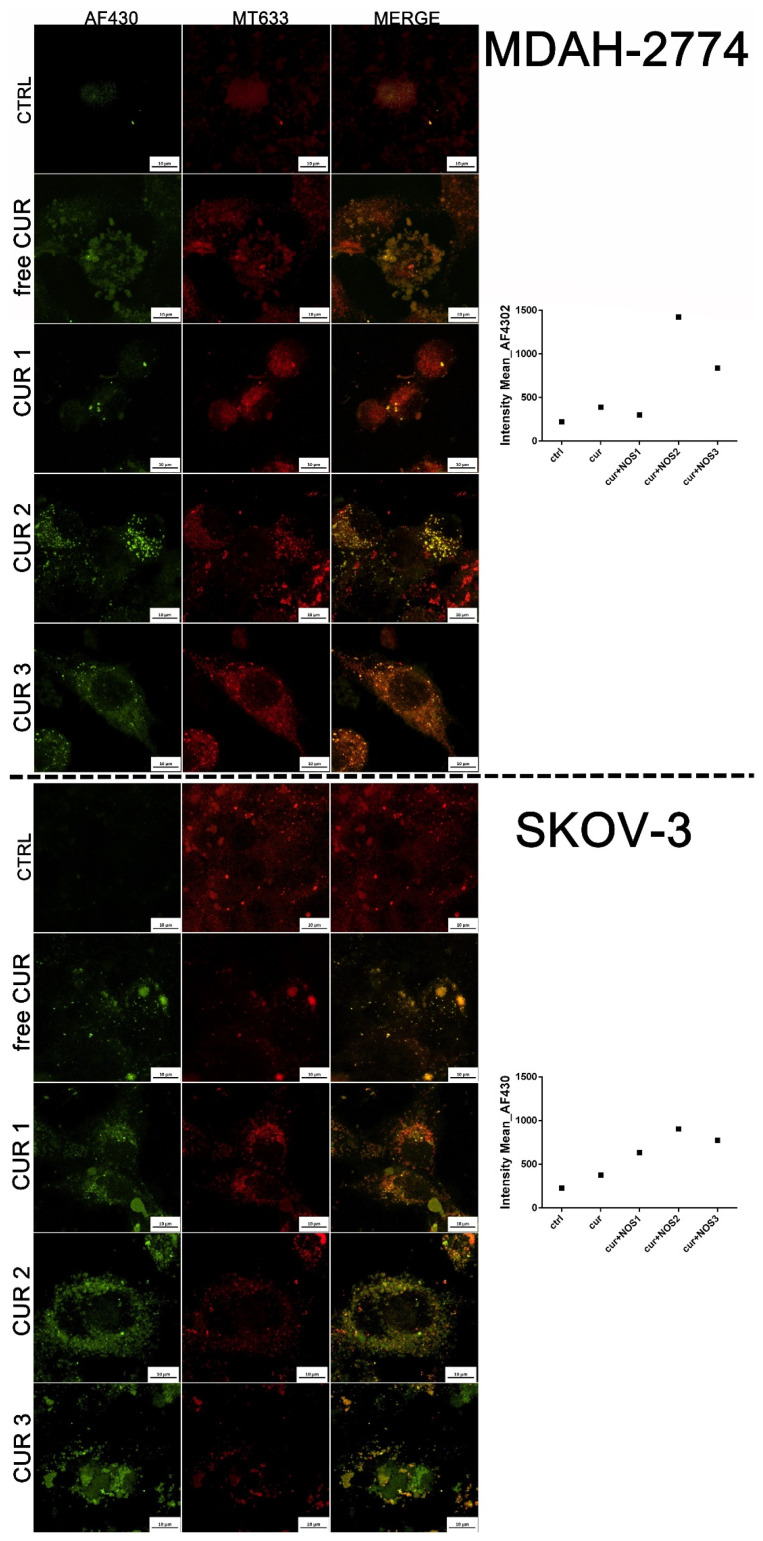
Confocal laser scanning microscopy (CLSM) visualization of mitochondria (in red, MT633) and intracellular localization of CUR (in green, AF430) after 24 h of incubation of SKOV-3 and MDAH-2774 ovarian cancer cells with free curcumin and curcumin loaded in the core–shell systems; graphs present mean intensity of fluorescence emitted from CUR.

The visualization of cells treated with CUR and CUR-loaded particles revealed that the entrapment of CUR in the designed core–shell system resulted in an enhanced cellular uptake, which was demonstrated through the increased intensity of the fluorescence emitted (Figure 4). The improvement in the cellular uptake was particularly high for MDAH-2774 cancer cells. For both cell lines, the delivery of the CUR 2 system was the most efficient, while, for the CUR 1 system, it was the weakest. The results of the CLSM study showed the colocalization of CUR with mitochondria, suggesting their role as a main cellular target for this phytotherapeutic.

## 3. Discussion

Natural compounds, including curcumin and resveratrol, offer a huge potential for the enhancement of ovarian cancer therapy due to multiple mechanisms of their anticancer activity. Numerous previous studies revealed i.a. that CUR induces apoptosis in ovarian cancer cells but not in normal cells [15] and RSV can trigger autophagy and subsequent apoptosis due to the induction of reactive oxygen species (ROS) generation [33]. Moreover, RSV can limit the invasive properties of ovarian cancer cells by affecting the expression of RNA messengers and microRNAs involved in cell locomotion, remodeling the extracellular matrix and rescuing the glycolysis-mediated inhibition of autophagy [34,35]. Taking into account these beneficial properties, we decided to use CUR and RSV as a payload for novel core–shell nanoparticles and evaluate their cytotoxic potential in human ovarian cancer cells. In our study, it was demonstrated that the half maximal inhibitory concentration (IC_50_) of free CUR and RSV was ca. 20 μM for MDAH-2774 cells. In contrast, the value of the SKOV-3 cell line strongly depended on the time of incubation. After the short incubation (24 h), the cytotoxicity of free CUR and RSV on SKOV-3 was low (IC_50_ reached ca. 90 μM), but, after 72 h, the cells became more sensitive to the treatment (IC_50_ was reduced to ca. 22 μM for RSV and 11 μM for CUR). These results show that the therapeutic efficacy of natural compounds still requires enhancement to exploit their potential and increase clinical applicability.

Our approach utilizes nanoparticles to improve the delivery of CUR and RSV. Such systems offer the possibility of being used for precise and selective drug delivery, partially due to the enhanced permeability and retention (EPR) effect occurring in the tumor tissue with a leaky vasculature [8,36,37]. The entrapment of CUR and RSV in various kinds of nanoformulations was proved to enhance the anticancer effect of both phytopharmaceuticals effectively. Dendrosomal nanocurcumin caused the downregulation of cell invasion and migration through the reduction of the expression of metalloproteinases (MMP-2 and MMP-9) [38]. The physicochemical and biological properties of RSV were improved by using various nanoparticles [36,39] or core–shell nanocapsules [40,41].

Core–shell nanocarriers for curcumin and resveratrol delivery were prepared to utilize newly devised and optimized—see [31,32]—methods by membrane-assisted processes for enhanced colloidal particle uniformity and tunability. It should be emphasized that a combination of composition and process parameters can yield systems of desired and previously designed parameters, especially in the fields of hydrodynamic diameter and polydispersity. Our systems were characterized by hydrodynamic diameters between 100 and 250 nm and relatively uniform sizes (spherical shapes confirmed by AFM)—features corresponding with the requirements for colloidal drug delivery systems. The core–shell structure of CUR and RSV nanocarriers, obtained in a similar manner, has been indirectly (by studies of dispersion forces’ interactions and zeta potential analyses) and directly (by means of XPS with ion sputtering) studied in previous studies [42,43]. Such findings clearly show the core–shell structure and indicate that a very thick, almost practically unimolecular, layer of HF-PE can sufficiently stabilize nanocarriers. Moreover, solubility parameter differences studies clearly show that the CUR-PLLA system is significantly more thermodynamically stable than the RSV-PLGA one, most possibly due to the lower molecular weight of PLLA building blocks (i.e., single lactic acid mer) compared with PLGA. Therefore, despite the lower molecular weight of RES, the system loaded with CUR is more thermodynamically compatible due to the higher “flexibility” of PLLA chains.

In our study, we evaluated the cytotoxic effects of both CUR and RSV loaded in three types of novel core–shell nanoparticles. The results obtained indicate significant differences between two types of cargo and three types of nanosystems. The cytotoxicity of the core–shell particles with CUR was significantly more effective in the inhibition of the mitochondrial activity of human ovarian cancer cells than in systems with RSV. For both types of cargo, nanocarrier systems nos. 2 and 3 were the most cytotoxic, which suggests the highest usefulness of such a platform for ovarian cancer therapy based on RSV or CUR. In a further step, a detailed in vitro evaluation of the specific molecular mechanisms of activity of CUR 2 and CUR 3 in ovarian cancer cells, followed by the in vivo study of their pharmacokinetics and pharmacodynamics, should be performed.

Another valuable direction of future research is related to the conclusions of various studies reporting that phytopharmaceuticals can be used as adjuvants of traditional chemotherapy, which may help to overcome the chemotherapy resistance of cancer cells [16,27,44,45,46]. The synergistic anticancer effect of a combination of CUR with paclitaxel against ovarian cancer was demonstrated in the in vitro and in vivo study [47]. Other research confirmed that RSV enhanced the cytotoxic effects of cisplatin [48] and doxorubicin [49] in ovarian cancer. Several studies have also focused on the application of nanoparticles for the co-delivery of chemotherapeutics and natural substances to improve ovarian cancer therapy. A great potential of the core–shell nanostructures for the delivery of curcumin and docetaxel [50] or curcumin and paclitaxel [4,51] to ovarian cancer cells was revealed. It has been reported that curcumin may act as a mediator of chemoresistance. The inhibition of a specific efflux pump can sensitize cancer cells to anticancer drugs [51]. Besides reversing drug resistance, the ability of natural compounds to protect normal cells against impairment induced by chemotherapy could be an additional benefit. Louisa et al. revealed that the delivery of cisplatin with nanocurcumin to an ovarian cancer rat model prevented cisplatin-induced renal and hematological toxicity [1]. Such combination therapy could be a very promising approach for further study, providing even more beneficial utilization of the potential of the core–shell nanoparticles designed.

## 4. Materials and Methods

### 4.1. Materials

Hydrophobic core-forming polymers, i.e., Poly(lactide-co-glycolide) (M_w_ =45 kDa; LA:GA = 50:50 and M_w_ = 45–70 kDa; LA:GA = 65:35) and poly(L-lactide) (PLLA) (Mw = 5 kDa) were obtained from Sigma-Aldrich (Burlington, MA, USA). Hydrophobic payloads (curcumin (CUR) and resveratrol (RES)) were from Archem. Hydrophobically functionalized polyelectrolytes (HF-PEs) and poly (ethylene succinate) were synthesized according to the methods described in [52,53], respectively. All solvents were of analytical grade and purchased from POCh (Avantor Performance Materials, Gliwice, Poland).

### 4.2. Preparation of the Core–Shell Nanoparticles

CUR and RSV-loaded core–shell nanoparticles were prepared according to procedures optimized and described in detail in [31,32], respectively. Briefly, an appropriate set (LDC-1, Micropore Technologies, Redcar, Cleveland, UK with NE-300 syringe pump, World Precision Instruments, Sarasota, FL, USA) comprising a vertical stainless steel stirrer, a glass cylinder for the continuous phase, an injection chamber for the dispersed phase, and a stainless steel (316) membrane (a pore diameter of 5 μm and a pore pitch of 200 μm (total open area: 0.0014 cm^2^) was prepared by filling it with appropriate solutions: the glass cylinder contained aqueous HF-PE (concentration—0.5 mg/mL), while the syringe contained the drug and hydrophobic polymers in acetone or an acetone/THF (1:1, *v*:*v*) mixture for RSV or CUR, respectively. The total concentration of hydrophobic polymers—poly(lactide-co-glycolide) (M_w_ = 45–70 kDa; LA:GA = 65:35) and poly(L-lactide) as well as poly(lactide-co-glycolide) (M_w_ = 45 kDa; LA:GA = 50:50) and poly (ethylene succinate) for CUR- and RES-loaded core–shell nanoparticles, respectively—was set as 10 mg/mL (2 mg/mL in final aqueous dispersion). The organic phase was introduced into the aqueous one through the membrane at a rate of 0.1 mL/min, corresponding to fluxes 71.4 mL/min/cm^2^, while the motor speed generated a shear stress of 18.7 Pa. After the whole intended amount of the organic phase was introduced, stirring was continued in the LDC-1 set for 10 min and the aliquots were transferred into a beaker. Stirring was continued for at least 12 h in order to assume organic solvent evaporation. Detailed information about the samples’ compositions is provided in Table 1.

### 4.3. Physicochemical Characterization

The size distribution (i.e., hydrodynamic diameter D_H_ and polydispersity index PdI) and zeta potential (ξ) of the studied core–shell nanoparticles were determined by dynamic light scattering (DLS) using a Zetasizer Ultra Red Instrument (Malvern Instruments, Malvern, UK) with a 10 mW He–Ne laser (λ = 632.8 nm). The detection mode was chosen automatically for an angle of 173° (i.e., noninvasive backscattering), 90°, or 13°. The analysis of the autocorrelation function was performed utilizing Dispersion Technology Software 8.10 from Malvern Instruments. All measurements were reproduced at least in triplicate, and the average value with standard deviation was taken for the intensity mean hydrodynamic diameter, polydispersity index, or zeta potential.

The concentrations of an active payload (curcumin and resveratrol) in colloidal dispersions were calculated utilizing the absorbance spectra of CUR and RSV recorded by spectrophotometric measurements (200–600 nm) with the use of a UV-3600 (Shimadzu, Kioto, Japan) double beam spectrophotometer (optical path—0.5 cm). Each sample was diluted fivefold with acetone prior to the measurement. The absorbance was recorded at 424 nm (CUR) or 326 nm (RSV), with the corresponding values for molar extinction coefficients equal to 50294 dm^3^mol^−1^cm^−1^ or 151.488 dm^3^mol^−1^cm^−1^, respectively, taken for the calculations. The determination of molar extinction coefficients by calibration curves in acetone/water (5:1, *v*:*v*) mixtures is shown in Figures S3 and S4 in ESI. The drug loading (*DL%*) and encapsulation efficiency (*EE%*) values were calculated according to Equations (1) and (2), respectively:

(1) $$DL\%=\frac{encapsulated\;mass\;of\;drug}{total\;mass\;of\;the\;nanoparticles}$$

(2) $$EE\%=\frac{encapsulated\;mass\;of\;drug}{total\;mass\;of\;the\;drug\;in\;the\;synthesis}$$

Atomic force microscopy was used for sample imaging. The fivefold-diluted (with double-distilled water) samples were dropped onto mica pieces, dried at room temperature, and analyzed using AFM apparatus (NaioAFM, Nanosurf company, Liestal, Switzerland)) in a tapping mode with a standard cantilever. The images obtained were analyzed using WSxM software (a freeware scanning probe microscopy software, ver. 5.0 develop 9) and are presented in Figures S1 and S2 in ESI.

### 4.4. Cell Culture

This study was performed on two human ovarian cancer cell lines: SKOV-3 and MDAH-2774. The SKOV-3 cell line is resistant to diphtheria toxin, cisplatin, and adriamycin. It was a kind gift from Professor Jakub Gołąb from the Department of Immunology, Center of Biostructure Research at the Medical University of Warsaw. The MDAH-2774 cell line was purchased from ATCC^®^ (Manassas, VA, USA). It is ovarian endometrioid adenocarcinoma, sensitive to cisplatin.

The cells were grown as a monolayer in a culture flask (Nunc, Roskilde, Denmark) in Dulbecco′s Modified Eagle′s Medium (DMEM, Sigma-Aldrich, St, Louis, MO, USA) with a high concentration of glucose (4500 mg/L) supplemented with 10% fetal bovine serum (FBS, Sigma-Aldrich) and 1% antibiotic solution (10,000 units penicillin and 10 mg streptomycin/mL (Sigma-Aldrich). The cultures were maintained at 37 °C in 5% CO_2_.

### 4.5. Cytotoxicity Evaluation

After growing, the cells were removed from the culture flask by trypsinization (trypsin 0.25% and EDTA 0.02%, Sigma-Aldrich), centrifuged, and resuspended in DMEM. Then, they were plated in 96-well microculture plates (Nunc) at a density of 5.0 × 10^4^ cells/well and allowed to attach for 16 h. Subsequently, the culture medium was replaced with 200 µL/well of resveratrol (RSV), curcumin (CUR), or their encapsulated formulations at varying concentrations (0.1–10 µM) in DMEM. The cells were incubated with the studied systems for 24 and 72 h at 37 °C with 5% CO_2_. Afterward, cell viability was assessed using the MTT (3-(4,5-dimethylthiazol-2-yl)-2,5-diphenyltetrazolium bromide) assay, which is the indirect method of measurement of a mitochondrial dehydrogenase activity, based on the detection of the ability of cells to reduce the tetrazolium dye MTT into its insoluble formazan. For this purpose, 100 µL/well of 0.5 mg/mL MTT solution in PBS buffer was added to the cells. After incubation for 2 h at 37 °C with 5% CO_2_, the generated formazan crystals were dissolved using 100 µL/well of acidified isopropanol (0.04 M HCl in absolute isopropanol). The absorbance at 560 nm was measured using a GloMax^®^ Discover Microplate Reader (Promega, Madison, WI, USA). The results were presented as the percentage of viable cells relative to untreated control cells with normal (100%) mitochondrial activity. The half maximal inhibitory concentration (IC_50_) values were also calculated using GraphPad Prism 9.5.1. software (GraphPad Software, La Jolla, CA, USA).

### 4.6. Confocal Laser Scanning Microscopy (CLSM) Visualization of Cells and Intracellular Localization of CUR

Cells were seeded on microscopic cover slides in 35 mm Petri dishes (Nunc) and incubated for 24 h to adhere. Then, the studied systems were added for 24 h. Afterward, cells were rinsed with PBS (BioShop Canada Inc., Burlington, ON, Canada), fixed in 4% formalin (10 min), washed 3× with PBS, and permeabilized with 0.5% Triton X-100 in PBS for 5 min. Then, cells were washed with PBS for 3 × 5 min and blocked with 1% bovine serum albumin (BSA, Sigma-Aldrich) in PBS for 1 h. After staining with Mitotracker (M22426, Invitrogen™), the cells were mounted in a fluorescence mounting medium (Fluoroshield™, Sigma Aldrich). For imaging, a super-resolution confocal laser scanning microscope (ZEISS LSM 980 with Airyscan 2, Zeiss, Oberkochen, Germany) was used. CUR was detected using a 430 nm excitation wavelength and 540 nm emission wavelength, and a mitochondria marker was detected by a 644 nm excitation wavelength and 665 nm emission wavelength. All experiments were performed in three independent repetitions. ZEN software (ZEN 3.10, Zeiss) was used for the quantification of the mean fluorescent signal.

### 4.7. Statistical Analysis

All measurements were carried out for *n* ≥ 9 for each group. The results were analyzed using GraphPad Prism 9.1.2 software (GraphPad Software, La Jolla, CA, USA) and expressed as mean ± SD. Data normality was tested using the Shapiro–Wilk test. Differences between the cytotoxicity of the analyzed particles and the untreated controls were assessed using parametric two-way analysis of variance (ANOVA) for multiple comparisons, with post-hoc Dunnett’s multiple comparisons tests. Differences between the free CUR or RSV and the core–shell systems were tested using parametric two-way ANOVA for multiple comparisons, with post-hoc Šídák’s (for RSV after 24 h) and Tukey’s (for RSV after 72 h) multiple comparisons tests. Differences between the study and control groups were considered statistically significant at *p* < 0.05.

## 5. Conclusions

The cytotoxicity of the novel core–shell particles with RSV on human ovarian cancer cells was rather slight, but the same systems loaded with CUR were significantly more effective in the inhibition of the mitochondrial activity of cells. For both natural compounds, type 1 nanocarriers (RSV I and CUR 1) were the least cytotoxic, which suggests the lowest usefulness of such a platform for ovarian cancer therapy based on RSV or CUR. Moreover, the results obtained showed that the MDAH-2774 ovarian cancer cell line was more sensitive to the treatment than the SKOV-3 cell line. The CLSM study showed an enhancement of cellular uptake of CUR after entrapment in the core–shell systems designed, as well as the colocalization of CUR with mitochondria. Further research focused on the biological effects of CUR 2 and CUR 3 should be conducted in order to provide detailed insights into their anticancer potential.

## Figures and Tables

**Table 1:** Characteristics of resveratrol- and curcumin-loaded core–shell nanoparticles.

System	HF-PE ^a^	Core Composition [mg/mL]	D_H_^f^ ± SD ^g^ [nm]	PdI ^h^ ± SD	c_payload_^i^ ± SD [μM]	c_payload_ ± SD [mg/mL]	DL ^j^ ± SD [%]	EE ^k^ ± SD [%]
**RSV I** ^l^	PSS-MA ^b^-g-C_16_OH(15%)	2 (PLGA ^d^)	144.0 ± 2.34	0.124 ± 0.006	324.1 ± 13.7	0.074 ± 0.003	3.7 ± 0.2	66.6 ± 2.9
**RSV II** ^m^	PSS-MA-g-C_12_OH(15%)	1.5 (PLGA) + 0.5 (PES ^e^)	146.2 ± 2.15	0.155 ± 0.021	395.1 ± 10.7	0.090 ± 0.003	4.5 ± 0.2	81.2 ± 2.2
**RSV III** ^n^	PSS-MA-g-C_16_OH(15%)	1 (PLGA) + 1 (PES)	129.7 ± 1.31	0.106 ± 0.028	398.3 ± 10.3	0.091 ± 0.003	4.5 ± 0.2	81.8 ± 2.1
**CUR 1** ^o^	PSS-MA-g-C_16_OH(15%)	2 (PLGA)	121.6 ± 1.75	0.091 ± 0.018	54.4 ± 1.8	0.020 ± 0.001	0.8 ± 0.1	80.2 ± 2.7
**CUR 2** ^p^	PSS-MA-g-C_16_NH_2_(15%)	2 (PLGA)	243.9 ± 16.3	0.152 ± 0.019	36.5 ± 1.2	0.013 ± 0.001	0.5 ± 0.1	76.8 ± 2.6
**CUR 3** ^q^	PAA ^c^-g-C_16_OH(15%)	2 (PLGA)	187.2 ± 4.19	0.074 ± 0.026	28.6 ± 1.0	0.011 ± 0.001	0.4 ± 0.1	84.3 ± 3.0

^a^ HF-PE—hydrophobically functionalized polyelectrolyte; ^b^ PSS-MA—Poly(4-styrenosulfonic-co-maleic acid); ^c^ PAA—poly(acrylic acid); ^d^ PLGA—*poly(lactic-co-glycolic) acid*; ^e^ PES—poly(ethylene succinate); ^f^ D_H_—hydrodynamic diameter; ^g^ SD—standard deviation; ^h^ PdI—polydispersity index; ^i^ c_payload_—concentration of the payload; ^j^ DL—drug loading; ^k^ EE—encapsulation efficiency; ^l^ the core-shell system no. 1 loaded with resveratrol; ^m^ the core-shell system no. 2 loaded with resveratrol; ^n^ the core-shell system no. 3 loaded with resveratrol; ^o^ the core-shell system no. 1 loaded with curcumin; ^p^ the core-shell system no. 2 loaded with curcumin; ^q^ the core-shell system no. 3 loaded with curcumin.

**Table 2:** Half maximal inhibitory concentration (IC_50_) calculated for core–shell nanoparticles loaded with resveratrol (I, II, III) and free resveratrol (RSV) on two ovarian cancer cell lines (SKOV-3 and MDAH-2774) based on the results of MTT assay after 24 h and 72 h.

Studied System	IC_50_ [µM]			
	SKOV-3		MDAH-2774	
	24 h	72 h	24 h	72 h
**Free RSV**	88.35	22.00	13.22	18.99
**RSV I** ^a^	5.653 × 10^39^	30.44	1.393 × 10^33^	78.23
**RSV II** ^b^	7.363 × 10^48^	30.93	48.20	17.24
**RSV III** ^c^	293.0	28.08	58.58	36.97

^a^ the core-shell system no. 1 loaded with resveratrol; ^b^ the core-shell system no. 2 loaded with resveratrol; ^c^ the core-shell system no. 3 loaded with resveratrol.

**Table 3:** Half maximal inhibitory concentration (IC_50_) calculated for core–shell nanoparticles loaded with curcumin (1, 2, 3) and free curcumin (CUR) on two ovarian cancer cell lines (SKOV-3 and MDAH-2774) based on the results of MTT assay after 24 h and 72 h.

Studied System	IC_50_ [µM]			
	SKOV-3		MDAH-2774	
	24 h	72 h	24 h	72 h
**Free CUR**	95.56	11.23	20.33	18.58
**CUR 1** ^a^	14.61	7.29	9.98	1.26
**CUR 2** ^b^	24.25	11.33	3.04	0.14
**CUR 3** ^c^	9.48	6.65	4.53	0.16

^a^ the core-shell system no. 1 loaded with curcumin; ^b^ the core-shell system no. 2 loaded with curcumin; ^c^ the core-shell system no. 3 loaded with curcumin.

## Data Availability

The datasets generated and/or analyzed during this study are available from the corresponding author upon reasonable request.

## Supplementary Materials

The following supporting information can be downloaded at https://www.mdpi.com/article/10.3390/ijms26010041/s1.
