# Supplementary material for: Cytotoxic Activity of Curcumin- and Resveratrol-Loaded Core–Shell Systems in Resistant and Sensitive Human Ovarian Cancer Cells
Source: Int J Mol Sci. 2024 Dec 24;26(1):41. doi: 10.3390/ijms26010041 (PMC11720041; doi:10.3390/ijms26010041)
Supplement: Supplementary file 1 [file ijms-26-00041-s001.zip › ijms-3286033-supplementary.pdf]

Electronic supplementary information

Cytotoxic Activity of Curcumin- and Resveratrol-Loaded Core–Shell Systems in Resistant and Sensitive Human Ovarian Cancer Cells

Joanna Weżgowiec [1], Zofia Łapińska [2], Łukasz Lamch [3], Anna Szewczyk [2,4], Jolanta Saczko [2,†], Julita Kulbacka [2,4], Mieszko Więckiewicz [1,*] and Kazimiera A. Wilk [3]

1. Physicochemical characterization of the core-shell nanoparticles.

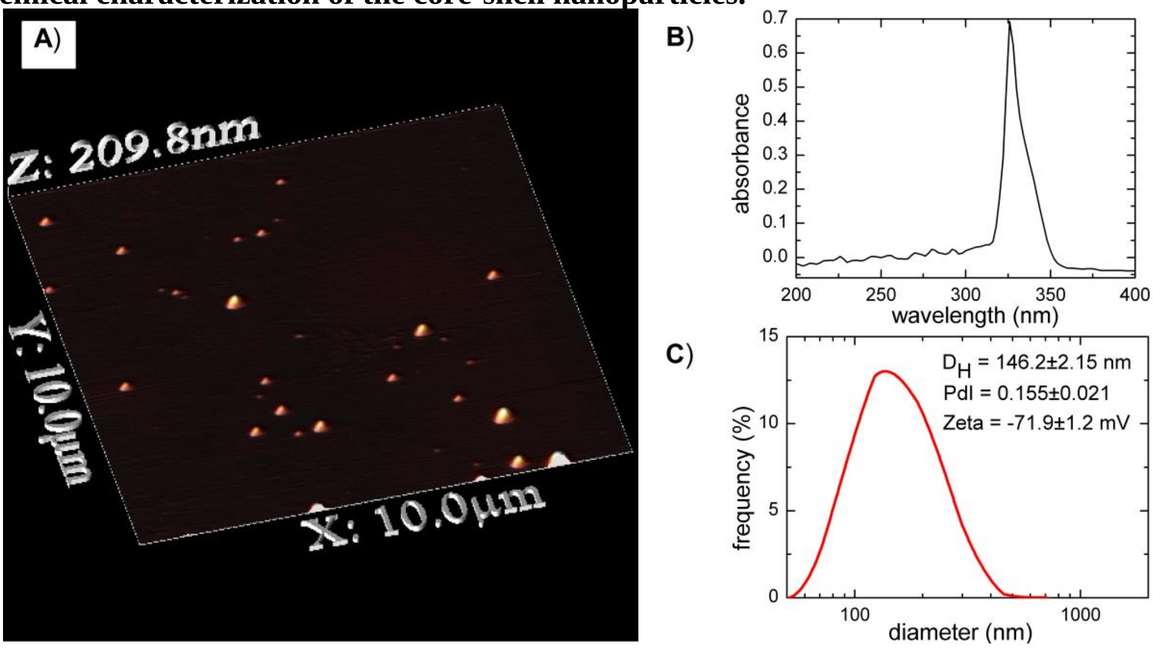

Figure S1. Characterization of RSV-loaded core-shell nanoparticles (see RSV II in Table 1): 3D AFM imaging – A), UV-Vis spectrum in acetone:water (5:1, *v:v*) – B) and DLS data (size distribution curve, the obtained values of D_H, PdI and zeta potential) – C).

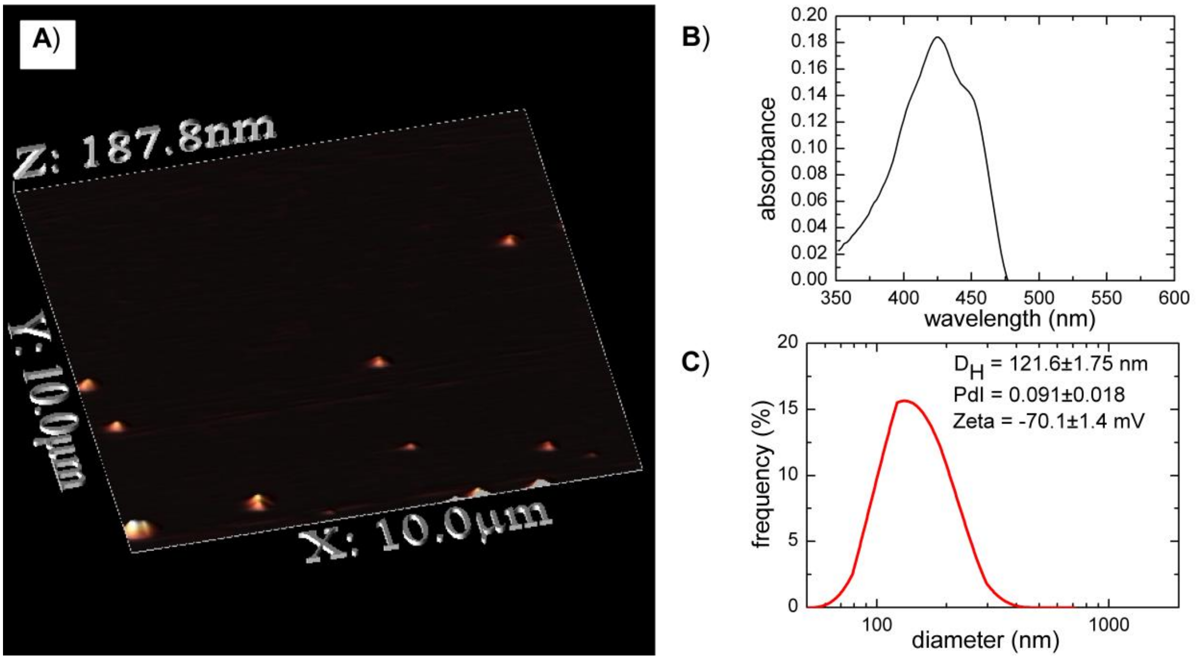

Figure S2. Characterization of CUR-loaded core-shell nanoparticles (see CUR 1 in Table 1): 3D AFM imaging – A), UV-Vis spectrum in acetone:water (5:1, *v:v*) – B) and DLS data (size distribution curve, the obtained values of D_H, PdI and zeta potential) – C).

2. Calibration curves for curcumin and resveratrol concentration determination in acetone:water (5:1, *v:v*) mixture.

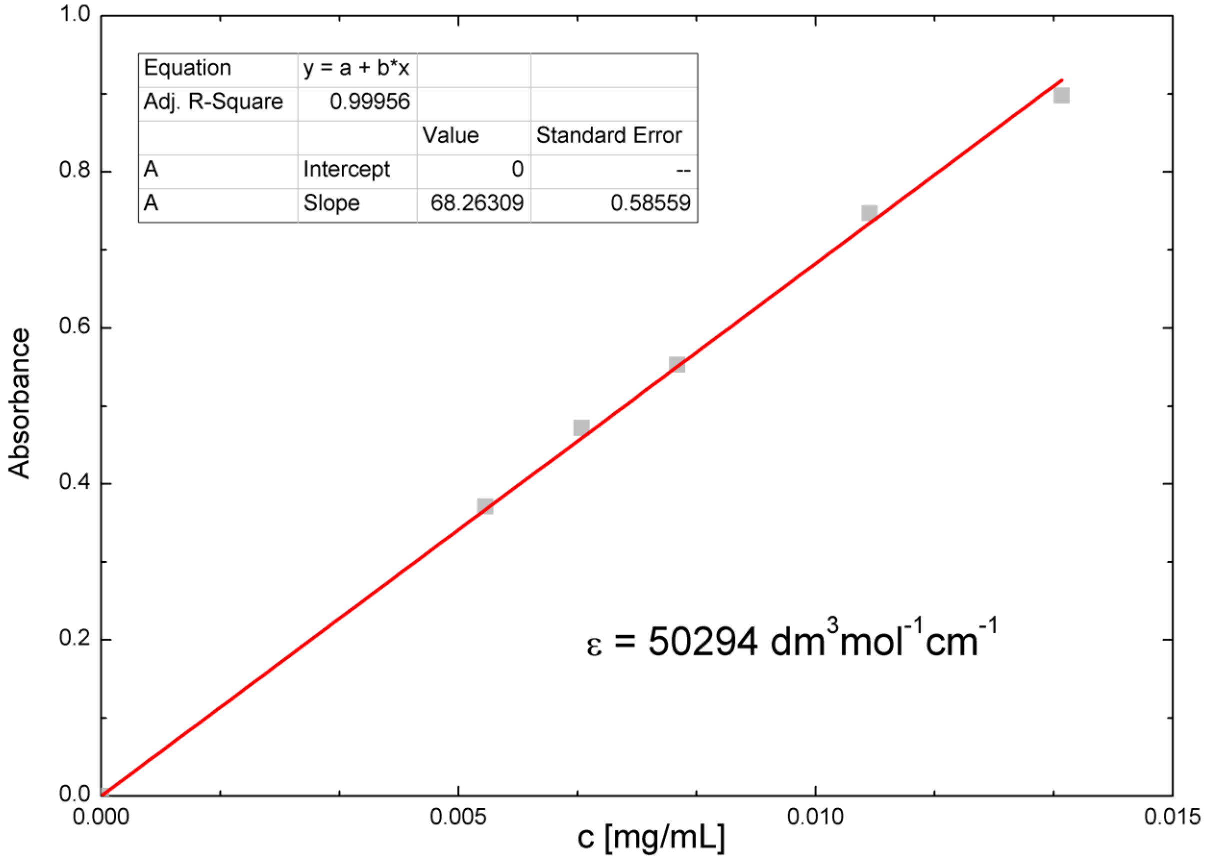

Figure S3. Curcumin calibration curve in acetone:water (5:1, *v*:*v*) mixture.

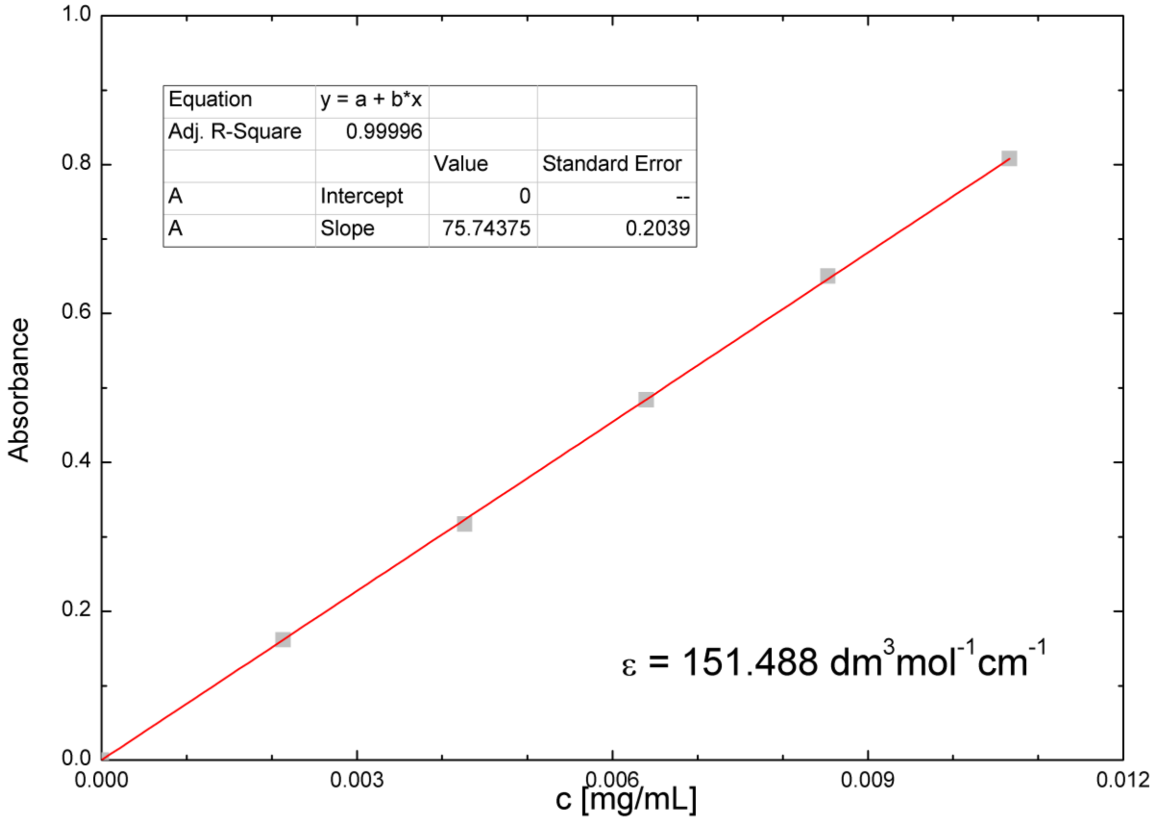

Figure S4. Resveratrol calibration curve in acetone:water (5:1, *v*:*v*) mixture.

3. Calculation of solubility parameters for appropriate solvents toward PES oligomers purification

Our studies comprise the use of the Y-MB approach (HSPiP (Ver. 5.1.08) software) for both polymers (i.e. Poly(lactide-co-glycolide) (PLGA) and poly(L-lactide) (PLLA)) as well as payloads (i.e. curcumin (CUR) and resveratrol (RSV)). It should be noted, that for calculations only the main polymer, i.e. hydrophobic core building one (poly(L-lactide) for CUR-loaded systems and poly(lactide-co-glycolide) (M_w =45 kDa; LA:GA =50:50) for RSV-loaded nanocarriers), is taken into account, but no plasticizer (poly(lactide-co-glycolide) (M_w =45–70kDa; LA:GA =65:35) for CUR-loaded nanocarriers and poly(ethylene succinate) for RSV-loaded systems) are taken into account. Solubility parameter components of dispersion (δ_D), polar (δ_P), and hydrogen bonding (δ_H) forces were calculated, while δ_H values were split into donor (δ_{HD}) / acceptor (δ_{HA}) counterparts:

$$\delta_H = \sqrt{\delta_{HD}^2 + \delta_{HA}^2} \quad (1)$$

Therefore, the formula for $\Delta\delta$, i.e. the difference between two substances (superscripts 1 and 2), follows the equation:

$$(\Delta\delta)^2 = 4\left(\delta_D^1 - \delta_D^2\right)^2 + \left(\delta_P^1 - \delta_P^2\right)^2 + 2\left(\delta_{HD}^1 - \delta_{HD}^2\right)\left(\delta_{HA}^1 - \delta_{HA}^2\right) \quad (2)$$

The latter extensions (equations 1 and 3) are particularly useful for miscibility of two large molecular weight components, e.g. formation of polymer blends, therefore are expected to constitute the useful extension for active payload-polymer matrix systems.

Table S1. Solubility parameter components and differences for CUR-PLLA and RSV-PLGA pairs.

Substance	Solubility parameter components				Differences
	δ_D [MPa$^{0.5}$]	δ_P [MPa$^{0.5}$]	δ_{HD} [MPa$^{0.5}$]	δ_{HA} [MPa$^{0.5}$]	$\Delta\delta$ [MPa$^{0.5}$]
CUR	20.1	8.1	7.3	5.2	15.0 (CUR-PLLA)
PLLA	16.1	13.3	16.7	12.4	
RSV	20.9	6.7	12.6	6.1	30.1 (RSV-PLGA)
PLGA	15.9	14.8	32.2	25.0	
