# Supplementary figures and images for: Cytotoxic Activity of Curcumin- and Resveratrol-Loaded Core–Shell Systems in Resistant and Sensitive Human Ovarian Cancer Cells
Source: Int J Mol Sci. 2024 Dec 24;26(1):41. doi: 10.3390/ijms26010041 (PMC11720041; doi:10.3390/ijms26010041)

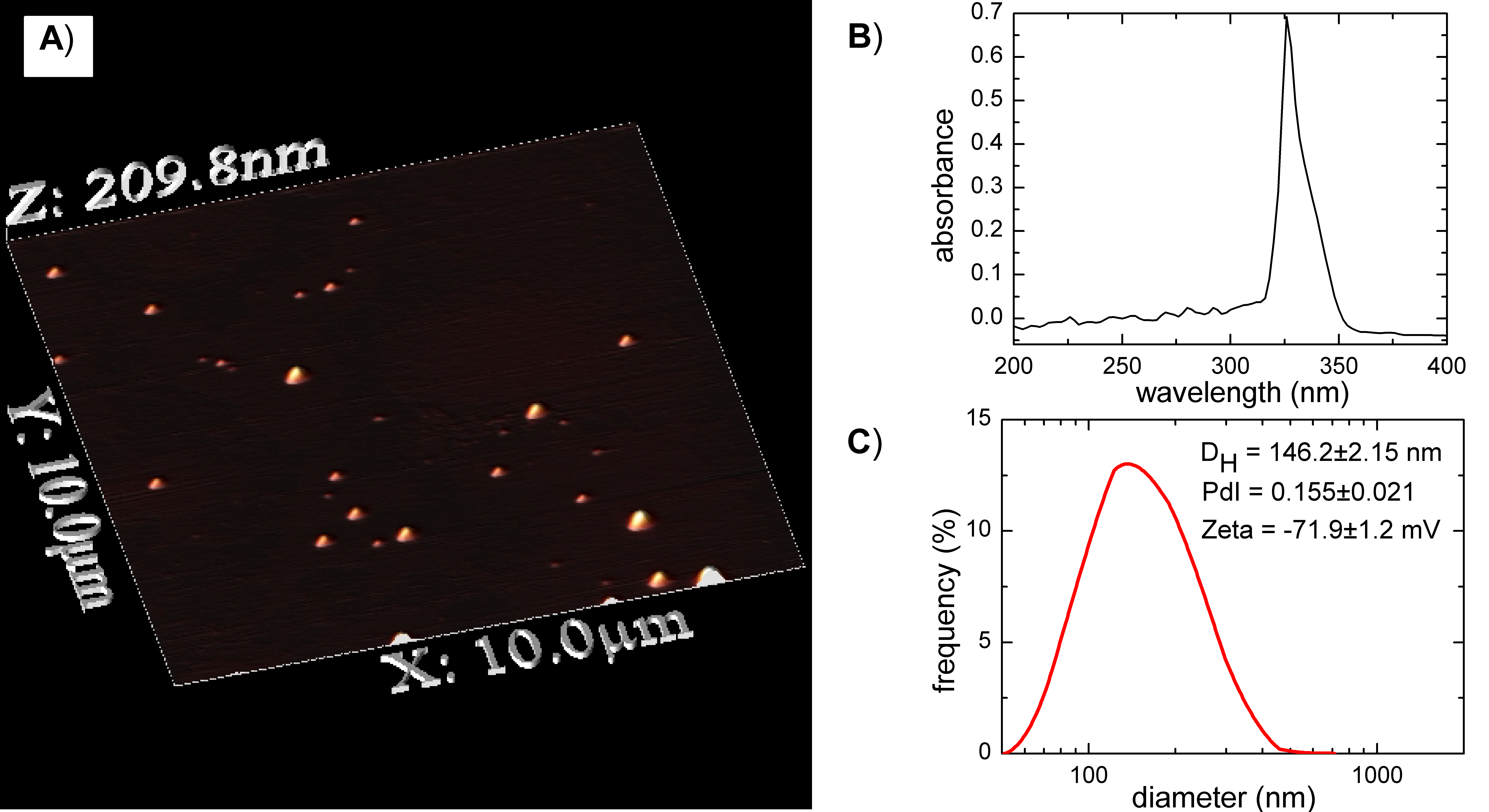

Supplement: Supplementary file 1 [file ijms-26-00041-s001.zip › Figure_S1_new.tif]

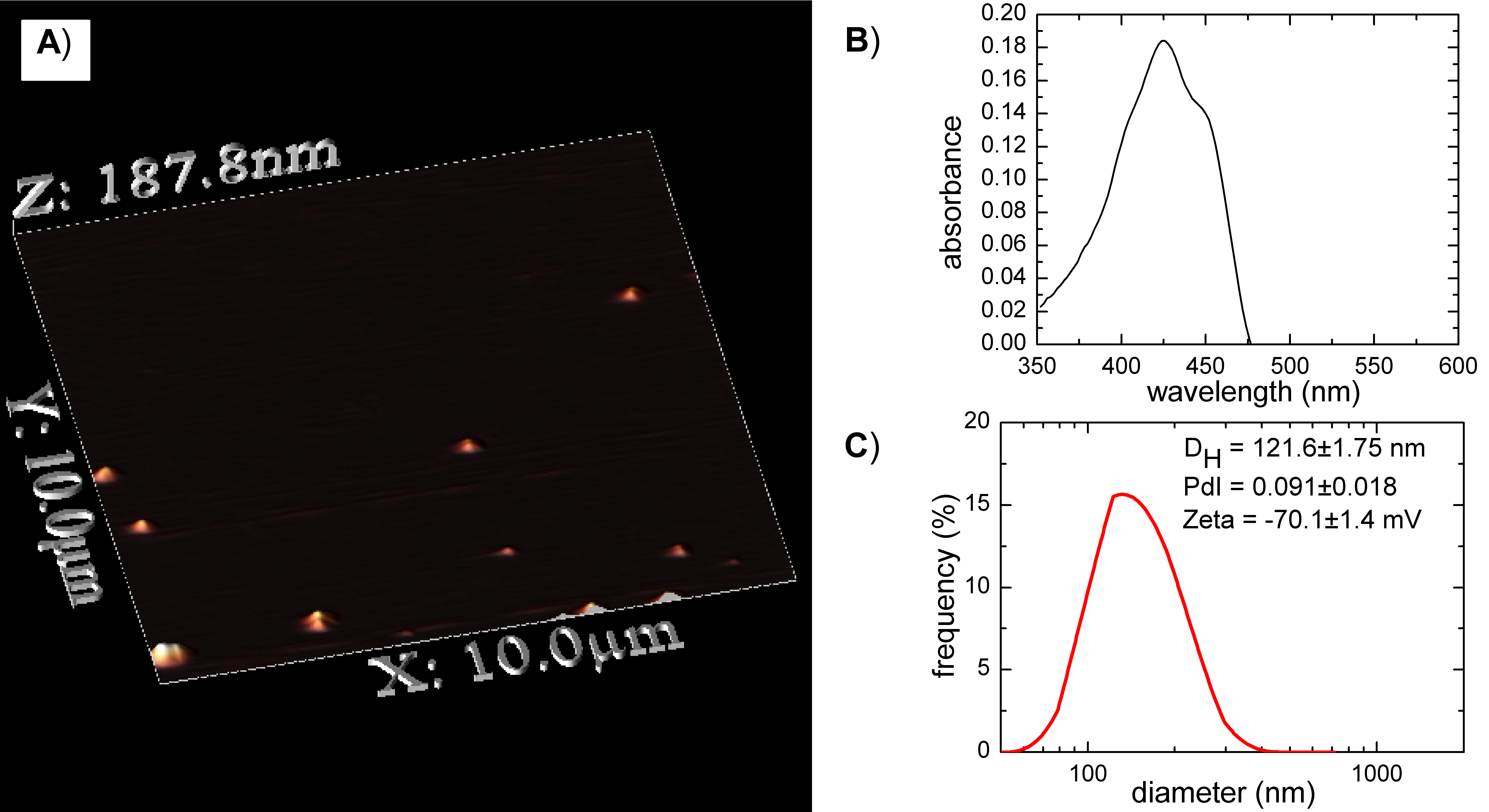

Supplement: Supplementary file 1 [file ijms-26-00041-s001.zip › Figure_S2_new.tif]
